# Changes in Physicochemical and Bioactive Properties of Quince (*Cydonia oblonga* Mill.) and Its Products

**DOI:** 10.3390/molecules28073066

**Published:** 2023-03-29

**Authors:** Katarzyna Najman, Sylwia Adrian, Anna Sadowska, Katarzyna Świąder, Ewelina Hallmann, Krzysztof Buczak, Bożena Waszkiewicz-Robak, Arkadiusz Szterk

**Affiliations:** 1 Department of Functional and Organic Food, Institute of Human Nutrition Sciences, Warsaw University of Life Sciences, Nowoursynowska 159c, 02-776 Warsaw, Poland; 2 Bioeconomy Research Institute, Agriculture Academy, Vytautas Magnus University, Donelaicio 58, 44248 Kaunas, Lithuania; 3 Department of Surgery, Faculty of Veterinary Medicine, Wroclaw University of Environmental and Life Science, Pl. Grunwadzki 51, 50-366 Wroclaw, Poland; 4 School of Health & Medical Sciences, University of Economics and Human Sciences in Warsaw, Okopowa 59, 01-043 Warsaw, Poland; 5 Center for Translational Medicine, Warsaw University of Life Sciences, Nowoursynowska 100, 02-797 Warsaw, Poland

**Keywords:** quince, *Cydonia oblonga*, physicochemical properties, tannins, polyphenol compounds, carotenoids, antioxidant potential

## Abstract

Quince (*Cydonia oblonga* Miller) is a plant that is commonly cultivated around the world, known for centuries for its valuable nutritional and healing properties. Although quince fruit are extremely aromatic, due to their high hardness and sour, astringent, and bitter taste, they are not suitable for direct consumption in an unprocessed form. However, they are an important raw material in fruit processing, e.g., in the production of jams, jellies, and juices. Quince fruits fall under the category of temperate fruits, so their shelf life can be predicted. Considering that technological processing affects not only the organoleptic properties and shelf life but also the functional properties of fruits, the aim of this research was to determine the impact of various types of technological treatments on the physicochemical and bioactive properties of quince fruit. In fresh, boiled, and fried fruits and in freshly squeezed quince fruit juice, basic parameters, such as the content of dry matter, moisture, soluble solids (°Brix), pH, total acidity, water activity, and color parameters (L*a*b*) were determined. The content of key bioactive ingredients, i.e., tannins, carotenoids, flavonoids, phenolic acids, and total polyphenols, was also determined, as well as the antioxidant activity of raw and technologically processed (cooked, fried, and squeezed) quince fruits. The conducted research showed that fresh quince fruit and processed quince products can be a very good source of bioactive ingredients in the diet, such as tannins (3.64 ± 0.06 mg/100 g in fresh fruit; from 2.22 ± 0.02 mg/100 g to 5.59 ± 0.15 g/100 g in products), carotenoids (44.98 ± 0.18 mg/100 g in fresh fruit; from 141.88 ± 0.62 mg/100 g to 166.12 ± 0.62 mg/100 g in products), and polyphenolic compounds (246.98 ± 6.76 mg GAE/100 g in fresh fruit; from 364.53 ± 3.76 mg/100 g to 674.21 ± 4.49 mg/100 g in products). Quince fruit and quince products are also characterized by high antioxidant properties (452.41 ± 6.50 µM TEAC/100 g in fresh fruit; 520.78 ± 8.56 µM TEAC/100 g to 916.16 ± 6.55 µM TEAC/100 g in products). The choice of appropriate technological processing for the quince fruit may allow producers to obtain high-quality fruit preserves and act a starting point for the development of functional products with the addition of quince fruit in its various forms, with high health-promoting values and a wide range of applications in both the food and pharmaceutical industries.

## 1. Introduction

Common quince (*Cydonia oblonga* Miller) is the only representative of the quince genus and belongs to the *Rosaceae* family, which also includes apple and pear trees [1]. Its place of origin is considered to be the western part of Asia, although it has now spread to other continents where it occurs both as a wild plant, planted in home gardens or parks as an ornamental, and is cultivated for its edible fruit [2]. The largest global producer and distributor is Turkey (supplying 25% of the global production), followed by China, Iran, Argentina, and Morocco (supplying 10% of the global production) [3,4]. Turkey’s production of quince fruit increased by about 66% between 2006 and 2020 [5].

In the middle latitudes of the northern hemisphere and in Poland, the flowering period of quince coincides with the flowering period of apples and falls at the end of May and beginning of June, while the fruit ripens relatively late—at the turn of September and even at the end of October, which is when the fruit is also harvested [1,4,6,7]. The fruit usually reaches a diameter of 3–5 cm, an average weight of 100–200 g (some varieties even weigh up to 1 kg), and has a shape that resembles apple or pear fruit, which is why they are often called “apple fruit” [1,4]. Ripe fruits are characterized by an intense, lemon-fruity smell and a golden-yellow color. They are covered with leathery skin and characteristic moss [2,4,6]. After cutting the fruit, the light-yellow flesh darkens very quickly, i.e., as a result of the oxidation of contained polyphenolic compounds, mostly under the influence of polyphenol oxidase and light [1,6,8,9].

Quince fruit is increasingly arousing the interest of scientists and consumers due to its wide pro-health effects, which are associated with hypoglycemic, anti-inflammatory, anticancer, antibacterial, anti-allergic, and anti-ulcer properties [7,10,11,12]. The medicinal properties of quince fruit have been known for years, and it is widely used in folk medicine. A traditional method of treating ailments such as gastrointestinal disorders, nervousness, insomnia, cough, fever, respiratory disorders, urinary tract diseases, peptic ulcer disease, diabetes [13], skin lesions [14], migraine, colds, flu [15], inflammatory bowel diseases [16], and conjunctivitis [17] is the use of various parts of the quince: seeds, fruit, roots, and leaves. The quince’s therapeutic potential is attributed to its strong antioxidant potential, which results from the presence of plant secondary metabolites, mainly polyphenols (flavonoids, quercetin, rutin, and kaempferol) [1,2,18] and carotenoids, vitamin C, citric acid, malic acid, and quinic acid, which are present in both the skin and the flesh of the quince [19,20]. These fruits also provide valuable minerals, e.g., potassium, calcium, magnesium, and sodium [13,21,22,23], as well as fiber in the form of pectins and tannins [24]. However, they are low in carbohydrates, protein, and fat [2,25].

The taste of quince fruit pulp depends on many factors, including the variety and degree of ripeness or date of fruit harvest; however, the fruit is usually hard, sour, and astringent, with an unpleasant bitter aftertaste, which makes it unsuitable for raw consumption [12,26,27,28,29,30]. However, the sensory characteristics of these fruits and the high content of organic acids, sugars, aromatic compounds, and pectins make them ideal for the production of various preserves [26,27,28,29,30]. Both in the home and under industrial conditions, quince fruits are an excellent raw material for the production of preserves, such as jams, marmalades, pastes, purees, or juices, compotes, tinctures, and liqueurs [7,27,29,30,31,32,33,34]. Apart from preserves, they are used as an addition to teas, yoghurts, lemonades, ice cream, jellies, or confectioneries, both in the form of fresh fruit and in various forms as juices, pulp, or dried fruit [19,25,31,35].

The physicochemical properties of the raw materials of fruit determine their intended use, i.e., their suitability for direct consumption, their technological suitability, and their processing potential, which affect the quality of the obtained products [36,37]. Fruit processing (including quince fruit), especially thermal treatment, such as cooking, frying, baking, or drying, can lead to changes in organoleptic characteristics (texture, consistency, color, and smell), as well as to changes in the content and profile of bioactive compounds, including vitamins, minerals, amino acids, pectins, dietary fiber, polyphenols, and carotenoids [27,29,30,31,32,33,34].

According to the literature, heat treatment increased the total acidity (from 1.18 ± 0.13% to 1.51 ± 0.13%), the content of total soluble solids (from 14.10 ± 0.16% to 85.40 ± 0.17), total sugars (8.13 ± 0.23% to 46.95 ± 0.18%), and reducing sugars (from 5.04 ± 0.13% to 27.88 ± 0.31) in various products (e.g., candy, jam, or dehydrated slices) compared to fresh quince pulp [38]. Compared to fresh quince fruit, the total acidity (14.5%), reducing sugars (9.2%), and total sugars (14.7%) increased as a result of processing, reaching 27.2%, 26.92%, and 32.2%, respectively [31]. In the studies by Mir et al. (2016) [38], the content of total polyphenols (67.44 GAE/100 g) and the antioxidant activity (approx. 70% DPPH) in products obtained as a result of heat treatment also increased in the cases of jam and dehydrated slices (up to 73.41 mg and 78.67 mg GAE/100 g for the total polyphenols, respectively, or up to approximately 84% and 83% for antioxidant activity, respectively).

Due to the growing interest in food of a natural origin and with valuable nutritional properties and the high therapeutic and health potential of quince, these fruits are gaining increasing popularity among food producers and consumers. Considering not only the undesirable sensory characteristics but also the seasonal availability of fresh quince fruits, the food market is mainly dominated by processed products from these fruits [32]. Therefore, the aim of this study was to determine the effect of various types of technological processing (i.e., cooking, frying, and juicing) that are commonly used in both industrial and home conditions to process quince fruit on the physicochemical and bioactive properties of fresh quince fruit and its preserves. This work is a continuation of the research conducted and published by our team on the effect of various drying methods on the physicochemical and bioactive properties of quince fruit [39].

## 2. Results and Discussion

### 2.1. Physicochemical Properties of Fresh Quince Fruits and Their Products

**Figure 1:**
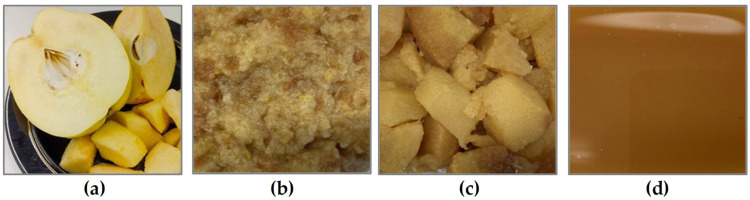
Changes in the appearance of fresh quince fruit (**a**) after technological processing: cooking in boiling water (**b**), frying in a pan without fat, (**c**) and juicing (**d**).

Changes in the appearance of fresh quince fruit (fresh) after technological processing, i.e., cooking in boiling water (cooked), frying in a pan without fat (fried), and juicing (juice), are presented in Figure 1.

The fresh quince fruits (Figure 1a) were of medium size (approximately 5–6 cm), oval in shape, resembling apples or pears, and had a lemon-yellow skin covered with a typical quince tuft. The flesh was not very juicy; it was slightly crumbly and light yellow in color. The fruit demonstrated an intense aroma that is characteristic of fresh quince. Cooked fruits (Figure 1b) were characterized by a thick, fairly uniform consistency (thick fruit mousse) and took on a light yellow, yellow, orange, and sometimes brown color while retaining a pleasant, typical, delicate quince aroma. Fried quince fruit (Figure 1c) had a compact consistency but crumbled easily. The product was characterized by a yellow-orange color and a quite perceptible fruity smell. Unlike the heat-treated products, quince fruit juice (Figure 1d) was characterized by a fairly uniform liquid consistency. It was naturally cloudy, without clearly visible fruit particles or residues, and had a dark orange and slightly brown color. It was characterized by a sweet and sour smell that is typical of fresh quince fruit. Technological and culinary processing (which are also carried out at home) changes the organoleptic characteristics, including texture, consistency, aroma, and color. Table 1 shows the color parameters in the L*a*b* color space of quince fruit and its products.

The highest (*p* ≤ 0.05) value for the L* color parameter was found in fresh quince fruit (82.11 ± 3.48), meaning that these samples were the brightest among all the samples analyzed. Heat treatment decreased this parameter by about 11.3%, meaning that the samples of boiled and fried quince were significantly darker (on average 72.83 ± 1.28) compared to the fresh fruit, although they did not differ from each other. The darkest sample was the freshly squeezed quince juice (56.47 ± 0.76), which was more than 30% darker than the unprocessed fruit. At the same time, the juice was characterized by the highest (*p* ≤ 0.05) value for the a* color parameter (13.99 ± 0.02), indicating the largest color shift towards red shades. Significantly lower values were recorded for the fresh and pan-fried fruit (no statistical differences between these samples), and the lowest (almost twice the a* value for juice) value was recorded for the boiled fruit (7.43 ± 0.01), indicating the lowest reddening of these samples. All the tested quince fruit samples were characterized by positive values of the b* parameter in the L*a*b* color space (the average value was 49.31 ± 6.89), meaning that they showed a color shift towards yellow. However, significant (*p* ≤ 0.05) differentiation was found in the intensity and saturation of the yellow color of individual samples. The lowest saturation of yellow shades was found in quince juice (41.61 ± 0.04), which in turn was characterized by the highest saturation of red color (13.99 ± 0.02). Higher values for the b* color parameter were found in the heat-treated fruits (cooked and fried), and the highest b* value was found in fresh, unprocessed quince fruits (58.76 ± 2.01). Therefore, fresh fruits were characterized by the highest yellow color saturation and the highest brightness compared to the processed quince products. The conducted research showed that quince is a type of fruit with a very strong tendency toward intense browning (BI). The browning index of fresh fruit was very high and amounted to 124.84 ± 15.08. Similar values (120.60 ± 7.73) were found in the pan-fried fruit. Indeed, the lowest BI was found in boiled fruit (89.78 ± 0.31), while the highest BI was found in freshly squeezed quince fruit juice (155.36 ± 2.92).

The high rate of browning and changes in the color of quince fruits toward darker shades under the influence of the applied treatment or exposure to light were confirmed in research by Guiné and Barroca (2014) [40] on the kinetics of the browning reaction of quince exposed to weather conditions. The authors showed that during 2 h of exposure, the browning index increased from 50 (measurements made immediately after cutting the fruit) to about 140 (after 2 h), with the largest changes occurring in the L* (70%), a* (83%), and b* (close to 100%) parameters within the first 30 min of exposure. This may explain the high browning index values obtained in this study.

According to the literature, the color of food products is influenced by many factors, including: conditions of transport and storage, during which the degradation of colored substances (particularly chlorophylls and carotenoids) occurs; processing (particularly thermal treatment); and enzymatic and non-enzymatic browning reactions [41,42,43]. Enzymatic fruit browning reactions are mainly due to the high activity of enzymes such as peroxidases and oxidoreductases, particularly polyphenol oxidase [8,9,44]. This enzyme catalyzes various oxidation reactions of the phenolic compounds present in quince, e.g., the oxidation of monophenols to diphenols and these to quinones, which then polymerize to dark-colored compounds [8,45]. Initiated in this way, the enzymatic browning process (a chain reaction) is difficult to stop and is ceased under natural conditions when the substances susceptible to it are completely browned [45,46]. In addition, quinones can also enter into non-enzymatic reactions with the phenolic compounds present in quince, leading to the formation of brown melanoidins [8,9,47,48], which are also formed in the late stages of the Maillard reaction as a result of the condensation of hexoses, fructans, amine components, or pyrroles [41,42,43,47]. In addition, the factors that may affect the color of fruit, including quince, are also the physicochemical characteristics of the fruit, such as its pH; total acidity; content of sugars, in particular reducing sugars that constitute Maillard reaction substrates; content of polyphenolic compounds; temperature; processing conditions; or time of exposure to light and atmospheric oxygen [40,49,50,51,52].

Considering that due to their undesirable taste (high hardness and astringency of the flesh), quince fruits are not eaten in a fresh, unprocessed form but are a valuable raw material in fruit processing, the impact of various technological processes on the physicochemical properties determines both the technological suitability of the fruit as well as its characteristics, properties, and the quality of the final products obtained from the fruit [36,37,53]. For this reason, the physicochemical characterization of quince fruit, basic parameters such as dry matter, water activity, soluble solids content (°Brix), pH, and total acidity, was determined in fresh quince fruit and quince fruit subjected to selected types of technological processing (cooking, frying, and juicing). The obtained results are presented in Table 2.

The lowest dry matter content was found in boiled quince (15.29 ± 0.38%) and quince juice (16.92 ± 0.07%), in which the dry matter content was lower than in fresh fruit (18.60 ± 0.16%), likely due to the loss of some ingredients during cooking and extrusion. The highest (*p* ≤ 0.05) content of dry matter was found in the fruit fried in a pan (21.90 ± 0.00%), probably due to the significant evaporation of water during this process.

In the studies conducted by Al-Zughbi and Krayem (2022) [54], fresh quince fruits were characterized by a lower content of dry matter (at the level of 15.73%), while they were higher and ranged from 21.54% to 26.79% [55] or between 18.60 and 27.10% in others [12]. According to the literature, differences in dry matter content may result from many factors, including the variety, date, conditions of harvesting, growth stage, and degree of ripeness of the fruit [12,54,55].

In the conducted research, fresh quince fruits were characterized by a moisture content of 81.40 ± 0.16%, and these obtained values were similar to the results of other authors. Rodrıguez-Guisado et al. (2009) [55] demonstrated a moisture content of quince flesh in the range from 73.11% to 78.46% (84.27% on average for five different varieties of quince); Rasheed et al. (2018) [56] demonstrated a moisture content from 83.94 ± 0.05% to 84.27 ± 0.11% (for fruit harvested in various locations in Pakistan); Mir et al. (2016) [38] found a value of 84.10%; and Szychowski et al. (2014) [12] found a value from 72.9 ± 0.5% to 81.04 ± 0.2% (for five different Spanish varieties), showing significant varietal differentiation.

In the conducted research, quince juice and quince fruit boiled in water showed a higher moisture content (83.08 ± 0.07% and 84.71 ± 0.38%, respectively), while fruit fried in a pan had the lowest values (78.10 ± 0.00%). However, in the available literature, no information was found on the moisture content of quince products subjected to such technological treatment. Both the dry matter content and the moisture content of raw fruit materials are important factors in determining their freshness, as well as determining the preservation of the quality of fruit preserves [53].

Regarding the water activity (Table 2), both the fresh fruit and the fruit products obtained as a result of technological treatment did not differ significantly. The average water activity of all tested samples was 0.98 ± 0.01; therefore, according to the literature, they should be classified as products with a high humidity and water activity (water activity > 0.85). And at the same time, they are susceptible to spoilage and the development of microorganisms [57]. High water activity and high moisture content affect the sensory characteristics of food products, providing them with the right texture, including softness or juiciness. However, high values of these parameters reduce the microbiological stability of food [58] and also favor enzymatic and non-enzymatic browning reactions, among other Maillard reactions, which may result in changes in taste and aroma and above all, in the color of products [49]; this was observed in this study.

The average pH value for the analyzed quince fruit samples was 3.91 ± 0.19, with the highest pH found in fresh fruit (4.19 ± 0.03) and significantly (*p* ≤ 0.05) lower pH values found in freshly squeezed quince juice (3.93 ± 0.01). The lowest pH values were found in the fruit samples subjected to heat treatment, i.e., the boiled and fried fruit (average 3.75 ± 0.03), with no effect of the type of heat treatment on this parameter. The obtained results are consistent with the results of Szychowski et al. (2014) [12], who demonstrated a pH of six different Spanish quince clones in the range from 3.96 ± 0.10 to 4.09 ± 0.10, Rodriguez-Guisaro et al. (2009) [55], who reported a pH in the range from 3.60 ± 0.2 to 3.84 ± 0.1 in the fresh fruit of five different varieties (also from Spain), or from the research of Curi et al. (2018) [35], who showed a pH from 3.67 to 4.46 in the fruit of ten varieties cultivated in a tropical region of Brazil. Lower values, ranging from 3.31 ± 0.03 to 3.44 ± 0.02, were reported by Rasheed et al. (2018) [56] in fresh quince fruit from various locations in Pakistan. In the available literature, no information was found on the pH of fruits subjected to heat-treatment such as boiling and frying, but this parameter is referred to in relation to preserves such as jams, marmalades [35], fruit pickles [31], jams with the addition of other fruits [32], or pastries with the addition of quince [59]. In the case of juices, there is a single datum in the literature, which mainly concerns the possibility of the inactivation of polyphenol oxidase from quince juice under the influence of thermal, high-pressure carbon dioxide, and ultrasonic treatments [8,9]. As the juices were produced differently, comparison of these results is not commensurate. The pH values of the non-thermally treated juices were 4.74 ± 0.23, which was higher than the juice obtained in this study [8,9].

Low pH values are usually accompanied by a higher total acidity [26,27], which was confirmed in this study. The fresh quince fruit with the highest pH value (4.19 ± 0.03) had the lowest total acidity (0.26 ± 0.00 g/100 g), while the fried fruit with the lowest pH (3.73 ± 0.01) had the highest (*p* ≤ 0.05) total acidity (0.55 ± 0.03 g/100 g) in terms of malic acid. Similar values (0.40 to 0.55%) were reported by Szychowski et al. (2014) [12] and Rodrıguez-Guisado et al. (2009) [55] (0.47–0.76%). Other acidity results available in the literature for fresh quince fruits were much higher and ranged from 0.81% to 1.00% [35], on average 1.18 ± 0.13% [38], or even from 1.11% to 1.25% [56]. According to the literature, the profile of organic acids shaping the total acidity included phytic, malic, quinic, citric and tartaric acids [12]. In the studies by Rodríguez-Guisado et al. (2009) [55], malic, tartaric, acetic, oxalic, ascorbic, and citric acids were recorded, while in the studies of Silva et al. [20,27,30], malic, quinic, ascorbic, citric, shikimic, oxalic, and fumaric acids were recorded. In the literature, there were differences in the mutual proportions of individual organic acids, with the dominant acids in most cases being malic acid [55], malic and quinic acid [30], or phytic and malic acid [12]. The differences in the profile of organic acids are explained by genetic factors (variety and genotype) and environmental factors (climatic and agronomic), as well as different extraction techniques and analytical procedures [12,20,27,30,55,56].

Based on the conducted research, significant (*p* ≤ 0.05) differences in the content of soluble solids (°Brix) in fresh and technologically treated quince fruits were shown (Table 2). The lowest total extract content was found in boiled quince and in fresh fruit, where it amounted to 12.25 ± 0.52% on average. A significantly higher extract was observed in quince juice (15.17 ± 0.29%) and fried quince (16.83 ± 0.58%).

Although no information was found in the available literature on the effect of thermal treatments, such as boiling and frying, on the extract content in quince fruit, the results of other authors for °Brix were consistent with those obtained in our own research for fresh fruit. For example, the °Brix for fruits of different quince varieties ranged from 1.33% to 10.33% [35] and from 11.57% to 14.70% [55]. A higher content of soluble solids was shown by Rasheed et al. (2018) [56] (13.94–14.22%), Mir et al. (2016) [38] (average 14.10 ± 0.16%), and Szychowski et al. (2014) [12] (15.10–17.20%), while Legua et al. (2013) [11] reported lower values, i.e., from 5.28% to 9.54%, for this parameter than in the current research. As in the case of other physicochemical indicators, e.g., pH, acidity, dry matter, or moisture content, differences in the °Brix in the literature are explained by many factors, including genetic, environmental, and particularly the degree of maturity of the fruit and the accumulation of natural sugars during fruit ripening [11,12,55]. According to the literature, the main sugars naturally accumulated in quince fruit are fructose and glucose, both in the pulp [12] and in the juice of quince fruit [55].

In the study, the content of soluble solids in the juice was higher (15.17 ± 0.29%) compared to the fresh fruit (12.50 ± 0.50%), which results from the applied technological processing. There are few studies on quince juice in the available literature. Iqbal et al. (2018, 2020) [8,9] showed slightly lower °Brix values (11.55 ± 0.22%), most likely due to the use of double-filtered juice in their experiment.

According to the literature, the physicochemical properties of fruit raw materials are a key factor in determining the final destination of fruit, i.e., its consumption in a natural or processed form [37]. Additionally, the quality of final fruit processing products depends to a large extent on parameters, such as the dry matter, moisture and sugar contents, total acidity, and pH, which determine the behavior and nature of the raw materials subjected to various types of technological processing and their potential for industrial processing [36,37].

A very important parameter for assessing the quality and intended use of fruit is the ratio of soluble solids to total acidity (°Brix/TA), which reflects not only the balance of sweetness and acidity [35] but also the degree of fruit ripeness [12,37]. Fruits with a simultaneously high sweetness and a low acidity are characterized by an optimal balance that is suitable for direct consumption [35]. According to some authors [27,35,36,37], the higher the °Brix value in fruits, the more desirable they are to consumers.

A high °Brix also indicates the ripeness of quince fruit, as 80% of this value is sugars that naturally accumulate during fruit ripening [37]. The fresh fruit maturity index in the study was 48.75 ± 2.36, which was higher than the index reported by Szychowski et al. (2014) [12] (from 30.6 ± 3.2 to 39.9 ± 5.1) or Rodrıguez-Guisado et al. (2009) [55] (from 18.04 ± 1.0 to 4.62 ± 1.5), proving a high degree of maturity of the tested fruits and their suitability for consumption. However, the direct consumption of unprocessed quince fruits is limited by, among others, a high pH, high astringency, bitterness (probably due to the high concentration of tannins), or the presence of other components that ultimately shape the characteristic flavor profile of quince fruit [24,54,55]. Due to the fact that quince fruits are hard, sour, bitter, and tart, they cannot be eaten raw; however, their chemical composition, sensory characteristics, and biological activity make these fruits a valuable raw material in food processing. They are widely used in the production of jams, marmalades, marinades, juices, and syrups, and they are also an excellent addition to cakes, desserts, honey, liqueurs, juices, teas, or wine as both fresh or dried fruit and in juice form [27,28,29,30,34,39,59,60,61,62].

### 2.2. Bioactive Properties of Fresh Quince Fruits and Their Products

**Figure 2:**
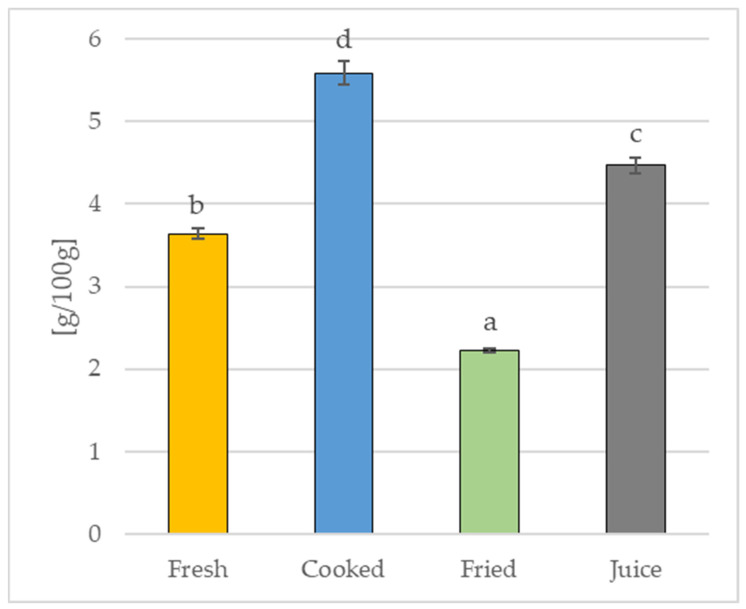
Tannin content in fresh quince fruit and its products. Mean values (*n* = 3) marked in bars by different letters differ significantly (Duncan’s test, *p* ≤ 0.05).

Figure 2 shows the content of tannins in the fresh quince fruit and its products, i.e., fruit boiled in water, fried in a pan without fat, and fresh quince fruit juice. The applied technological processing significantly (*p* ≤ 0.05) changed the content of tannins in quince fruit. The lowest content of these components was found in the fried fruit (2.22 ± 0.02 g/100 g), proving that heat treatment significantly decreased (by approx. 39%) the tannin content compared to the fresh fruit (3.64 ± 0.06 g/100 g). In turn, the remaining fruit samples were characterized by a significantly (*p* ≤ 0.05) higher content of these phytonutrients. The content of tannins in the freshly squeezed juice was 4.47 ± 0.09 g/100 g, while in fruit boiled in water it was as much as 5.59 ± 0.15 g/100 g, indicating that the concentration of these components was, respectively, approx. 23% and 53% higher than in the fresh, unprocessed quince fruit.

Unfortunately, the available literature does not contain data on the content of tannins in either boiled or fried quince fruit or in quince juice. There are also few studies on the content of tannins in quince in general [24,39]. According to Djila et al. (2021) [24], the content of tannins in quince fruits ranged from 7.3% to 9.7%, which is a higher range than in this study. However, the authors determined the content of tannins using other methods (precipitation and colorimetry) and not by titration, as was the case in our experiment; in turn, this causes great difficulties in comparing these results. Regarding the recently published results of research on the effect of various drying methods on the bioactive properties of quince fruit [39], in which we demonstrated significant differences in the content of tannins in convectively dried fruit (from 5.08 ± 0.4 mg/100 g to 6.85 ±0.61 mg/100 g) and sublimation (9.74 ± 0.05 mg/100 g), it should be stated that the effect of various types of technological processing on the content of tannins in quince fruit is not unambiguous and obvious. This is most likely due to the large variety of compounds classified as tannins, as well as the diversity of chemical properties of these bioactive ingredients [24,63,64].

In the literature, various polyphenolic compounds with molecular weights ranging from 500 to even 3000 Da are classified as tannins [63]. These compounds, thanks to a large number of functional groups (e.g., hydroxyls), bind permanently to proteins [65], polysaccharides such as cellulose, pectin, or starch [63], metal ions, or alkaloids [66], forming water-insoluble and non-degradable complexes [67]. Some of these tannins may undergo a hydrolysis reaction in an aqueous environment under the influence of various factors, such as acids, bases, some enzymes, or a high temperature, which is a generally accepted criterion in the literature for dividing these compounds into hydrolyzing and non-hydrolyzing tannins [63,64,67].

Hydrolyzing tannins (which usually occur in smaller amounts in plants) include mainly gallotannins, i.e., sugar esters (mainly glucose) of gallic acid or its depside form, and ellagitannins, i.e., derivatives of m-bigallic, trigallic, and hexahydroxydiphenic acids undergoing lactonization to ellagic acid [64]. As a result of their hydrolysis, the corresponding acids and glucose are formed [63]. On the other hand, non-hydrolyzing (condensed) tannins, also called proanthocyanidins (which are widely distributed in plants), include derivatives of flavonoids with a catechin structure, polycondensation products of flavan-3-ols or flavan-3,4-diols which do not contain sugar residues [67].

The general concentration and the mutual proportions of tannins (hydrolyzing and non-hydrolyzing) are varied. They naturally accumulate mainly in the cell sap, vacuoles, and cell walls of plants, protecting them against pathogens, herbivores, and adverse environmental influences [64]. Their high concentration in raw fruit (which decreases during ripening) gives the fruit a specific, sour-bitter taste [63,64]. Tannins are also found in products such as wine, beer, or some fruit juices; the appropriate share of tannins shapes their characteristic astringent taste, making the drinks too bland (in the case of insufficient content) or too sour and bitter (in the case of too high concentration of these relationships) and unpleasant in taste [68].

Tannins are primarily known for their astringent properties, which are widely used not only in the cosmetics or pharmaceutical industries (in ointments, gels, or creams with a pore-narrowing effect, in preventing seborrhea and soothing irritation, and in reducing itching, burning, or swelling) but also in medicinal products or dietary supplements, in particular for the treatment of diarrhea [63]. In addition, thanks to their astringent properties, binding proteins, and polysaccharides of microbial cell walls, tannins have antiviral, antibacterial, and antifungal activities [24,69,70]. Their ability to bind alkaloids is used, e.g., in the treatment of food poisoning [69,71]. In turn, their metal complexation may inhibit the activity of some enzymes (such as lipoxygenase, hyaluronidase, protein kinase C, or angiotensin-converting enzyme), exerting an anti-inflammatory effect [72]. Finally, as polyphenolic compounds that show strong antioxidant properties, they can prevent various chronic diseases with a free radical background [24,63,67] and have anti-cancer or anti-proliferative effects (e.g., the ellagic acid released during the hydrolysis of ellagitannin has a strong anti-carcinogenic effect) [63,73].

The conducted research showed a high concentration of tannins in the fresh quince fruit and a significant effect of the applied technological treatment on the content of these bioactive components in the quince fruit products. These results, and the results of our previous research [39], indicate that quince consumed in various forms (boiled, fried, dried, freeze-dried, or in the form of juice) can be a very good dietary source of tannins. However, it should be noted that the excessive consumption of these ingredients may cause side effects. If tannins form complexes with various macronutrients, it can lead to a decrease in the nutritional value of food, e.g., they can reduce protein digestibility, hinder the digestion and absorption of nutrients, limit the absorption of vitamins (e.g., vitamins A and B12) or mineral compounds (e.g., iron), and increase the excretion of certain cations, amino acids, or proteins [63]. Consuming tannins in excessive amounts can even lead to damage to the gastrointestinal mucosa [74]. However, it should be emphasized that the adverse effects occur only in the case of a high concentration of tannins in the diet, and the negative effects of tannins should not be observed with their average consumption in a balanced diet that is rich in proteins and minerals. Therefore, given the high content of tannins in quince fruit, special attention should be paid to these aspects. 

**Figure 3:**
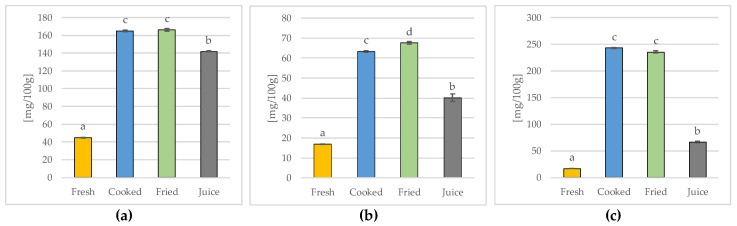
The sum of identified and quantified carotenoid (**a**), flavonoid (**b**), and phenolic acid (**c**) contents calculated as the sum of individual phenolic components identified by HPLC assay. Mean values (*n* = 3) marked in bars by different letters differ significantly (Duncan’s test, *p* ≤ 0.05).

As can be seen in Figure 3a, the tested quince fruits were characterized by a high content of carotenoids, and the technological treatment significantly changed both the content and profile of these compounds (Table 2).

The sum of determined carotenoids (Figure 3a) ranged from 44.98 ± 0.18 mg/100 g of the product (in the case of fresh fruit) to 166.12 ± 0.62 mg/100 g in fried fruit. The heat-treated fruits, i.e., the boiled and fried fruits, did not differ in terms of the sum of determined carotenoids (average 165.53 ± 1.13 mg/100 g), unlike the freshly squeezed quince fruit juice (141.88 ± 0.62 mg/100 g).

Table 3 presents the content of the determined carotenoid compounds in fresh and technologically processed (i.e., cooked, fried, and juiced) quince fruits. Among the five determined carotenoid compounds, chlorophylls (a and b) and β-carotene were present in the highest concentration in unprocessed quince, lutein was present in a lower concentration, and zeaxanthin was present in the lowest concentration. The results of these studies were published in our last article [39], and in the current study, they were used for comparative purposes. The technological treatment did not fundamentally change this tendency. However, the content of individual carotenoids significantly increased. In terms of the β-carotene content (which was present in the highest concentration in all products), the heat-treated quince fruits (boiled and fried) did not differ significantly from freshly squeezed fruit juice (average 54.38 ± 0.01 mg/100 g). Additionally, the boiled and fried fruits did not differ in terms of zeaxanthin content (average 5.56 ± 0.00 mg/100 g), which was present in the lowest concentration. However, differences were noted for chlorophyll a, chlorophyll b, and lutein. The average content of these bioactive ingredients in the heat-treated fruits was 48.45 ± 0.77 mg/100 g, 43.63 ± 0.77 mg/100 g and 13.50 ± 0.38 mg/100 g, respectively, and was significantly (*p* ≤ 0.05) higher than the in juice (by 22.1%, 17.8%, and 37.7% for chlorophyll a, chlorophyll b, and lutein, respectively).

There is little data in the literature on the carotenoid content in quince fruits, and no information has been found on the impact of various technological processes (i.e., cooking, frying, or squeezing juice) on the carotenoid content and profile in various quince products and preserves. Only the results of Ponder and Hallmann (2017) [75] confirm the trends obtained in this study. The authors showed that among the determined carotenoids, chlorophyll a prevailed in both the skin and in the pulp of quince, followed by chlorophyll b. Zeaxanthin and lutein were present in the lowest concentration. We obtained a similar relationship in our previous studies on the effect of various drying methods of quince fruit on the content of carotenoids [39]. Fruits dried by convection (in various thermal conditions) and freeze-dried fruits significantly differed in both their content and profile of carotenoid compounds, but in all dried samples, the dominant carotenoids were β-carotene, chlorophyll a, and chlorophyll b.

According to some authors, processing fruits and vegetables may lead to an increase in their carotenoid content after heat treatment [76], including cooking, steaming [77,78,79,80], drying, or smoking [81]. Borges et al. (2019) [79] showed that heat treatment, cooking in particular, can improve the release of bioactive compounds and increase the value of carotenoids in bananas. Ponder et al. (2021) [81] showed that the content of β-carotene in paprika may increase as a result of changes in the balance between β-carotene and its cis-β isomer during heat treatment. These authors also showed an increase in the content of lutein and xanthophylls (mainly due to changes in the concentration of cryptoxanthin and its isomer, β-cryptoxanthin) under the influence of various drying methods or smoking processes [81]. Higher contents of carotenoids were found by Bernhardt and Schlich (2006) [77] and Gliszczynska-Swiglo et al. (2006) [78] in cruciferous vegetables, Burmeister et al. (2011) [82] in potatoes, and Gunathilake et al. (2018) [80] in green leafy vegetables. The authors explained the increase in the concentration of carotenoids in cooked plants by increasing their availability [81], improving carotenoid extraction due to the breakdown of cellulose in the plant cell wall, and denaturing protein–carotenoid complexes [76,83] or protein–xanthophyll complexes, Burmeister et al. (2011) [82], under the influence of heat treatment.

Significant differences in both the composition and the profile of phenolic compounds (such as flavonoids and phenolic acids), determined by HPLC assay, were found in fresh quince fruits subjected to various types of technological processing. The obtained results are presented in Table 4.

The sum of determined flavonoids (Figure 3b) ranged from 16.92 ± 0.13 mg/100 g of the product (in fresh fruit) to 67.49 ± 0.66 mg/100 g (in fried fruit), and all tested samples differed statistically (*p* ≤ 0.05). In the case of heat-treated fruits, a significantly higher concentration of flavonoids was found in the fried fruits. In turn, freshly squeezed quince fruit juice contained a total of 40.07 ± 1.91 mg/100 g of these bioactive ingredients.

Epigallocatechin was the dominant flavonoid in both fresh, juiced, and heat-treated quince fruit, with the highest content found in fried quince (37.00 ± 0.78 mg/100 g), and lower contents (by approx. 18.5%) found in cooked fruit. Freshly squeezed quince juice contained almost three times less epigallocatechin than thermally treated fruits. In technologically treated quince fruits, a high content of rutinoside-3-*O*-quercetin was also found, with the highest (*p* ≤ 0.05) content found in fruits boiled in water (17.88 ± 0.81 mg/100 g) and a content approximately 8.8% lower found in the fried fruit and in juice (average 16.31 ± 0.72 mg/100 g). Additionally, in the case of catechin, the highest content was recorded in boiled quince (11.82 ± 0.25 mg/100 g), with significantly lower (*p* ≤ 0.05) contents found in fried and freshly squeezed juice by approx. 10.4% and 32.8%, respectively. In all the analyzed samples of quince fruit, quercetin was present in the lowest concentration. The heat-treated fruits, i.e., those that were boiled and fried, did not differ in their quercetin content (average 3.49 ± 0.10 mg/100 g), while the content of this component in the juice was half, 2.30 ± 0.07 mg/100 g.

In the available literature, there are few studies on the content of flavonoids in quince fruit, and there is also no information on the profile of these compounds in fruits subjected to various types of technological processing. In the studies conducted by Stojanović et al. (2017) [84], the total content of flavonoid compounds in acetone extracts from the peel and pulp of fruits in various quince species ranged from 41.23 to 63.41 mg CE/100 g dm. (in the skin) and from 17.28 to 44.65 mg CE/100 g f.w. (in the pulp), which was similar to the values obtained in this study, with the authors showing a higher content of flavonoids in the peel than in the flesh of the fruit. Similar values were also obtained Ponder and Hallmann (2017) [75], both for the sum of determined flavonoids (34.69 ± 1.95 mg/100 g) and for rutoside-3-*O*-quercetin (8.57 ± 0.54 mg/100 g) and quercetin (5.86 ± 0.50 mg/100 g) in fresh quince fruit. The tendencies observed in these studies (i.e., rutoside-3-*O*-quercetin appearing as the dominant flavonoid and definitely lower concentrations of quercetin and catechin) were also found by Essafi-Benkhadir et al. (2012) [85]. The contents of rutoside-3-*O*-quercetin, quercetin, and catechin in the studies by these authors were 47.21 ± 4.56 mg/100 g, 7.01 ± 2.92 mg/100 g, and 5.07 ± 2.15 mg/100 g, respectively, and these results concerned the skin of the quince fruit. Similarly, in studies conducted on the pulp and skin of quince fruit, Wojdyło et al. (2013) [7] showed similar values for rutinoside-3-*O*-quercetin and catechins in the pulp (5.10 mg/100 g and 1.09 mg/100 g, respectively) and definitely higher contents in the skin of quince fruit (20.28 mg/100 g and 37.85 mg/100 g, respectively). Additionally, in studies on the effect of various methods of drying quince fruit, dominant shares of epigallocatechin and rutinoside-3-*O*-quercetin in the determined flavonoids were shown, and definitely (2-fold) lower shares of catechin and quercetin [39] were found. However, in the literature, no data were found on the effect of cooking, frying, and juicing on the content of flavonoids in quince fruit.

Regarding the determined phenolic acids (Figure 3c), the sum of these compounds ranged from 16.88 ± 0.16 mg/100 g of the product (fresh) to 242.97 ± 1.25 mg/100 g (cooked), whereas the quince subjected to heat treatment (fried and boiled) did not differ from each other (average 239.22 ± 4.38 mg/100 g). The quince fruit juice contained a significantly lower (over 3.5 times) content of total phenolic acids (66.18 ± 1.49 mg/100 g) compared to the heat-treated fruit.

As can be seen in Table 4, the dominant phenolic acid in all analyzed fruit samples was chlorogenic acid (approx. 73% in juice and 89% in fried fruit). On the other hand, the smallest share had ferulic acid, which was only less than 1% (in fried fruits), approximately 2% (in fresh and cooked fruits) and slightly more than 3% (in juices). Regarding the *p*-coumaric acid content, no differences were found between the technologically treated fruit samples (average 6.38 ± 0.33 mg/100 g). The greatest differences were noted for gallic and caffeic acids. While their content was the highest (*p* ≤ 0.05) in boiled quince (15.71 ± 0.12 mg/100 g and 9.86 ± 0.10 mg/100 g, respectively), there were differences in the fried quince and quince juice. The highest contents of these compounds were detected in boiled quince (15.71 ± 0.12 mg/100 g and 9.86 ± 0.10 mg/100 g, respectively). In the case of gallic acid, a significantly (*p* ≤ 0.05) lower content was found in fried quince (14.68 ± 0.07 mg/100 g), and the lowest content (almost three times lower than in boiled quince) was found in the juice (5, 63 ± 0.11 mg/100 g), while in the case of caffeic acid, the juice contained more than twice the content (4.02 ± 0.04 mg/100 g), and the fried quince contained more than three times (2.91 ± 0.06 mg/100 g) less of this ingredient compared to the boiled quince (9.86 ± 0.10 mg/100 g).

Although there are studies in the literature on phenolic acids in quince fruits, they usually compare the profile of these compounds in the flesh and skin of different varieties [7,12,21,26,29,85] or in final quince products, e.g., quince jams with the addition of other fruits [32], sweet liqueurs [38], tinctures [34], juices from various quince varieties [33], or in fruits dried using various methods [38,39]. There are no data on the effect of technological treatment on phenolic acids, and the results existing in the literature fall within very wide limits. Some of them differ, while others are similar to the results in this work and show a similar trend in the share of individual components. For example, in the studies by Ponder and Hallmann (2017) [75], the content of phenolic acids was almost twice as high (34.69 ± 1.95 mg/100 g) in fresh fruit as the content found in these studies (16.88 ± 0.16 mg/100 g). The results of these authors also differ in the case of individual phenolic acids, e.g., for chlorogenic (3.27 ± 0.24 mg/100 g), caffeic (3.84 ± 0.33 mg/100 g), and *p*-coumaric (20.75 ± 2.66 mg/100 g) acids. They also demonstrated the highest share of *p*-coumaric acid and not chlorogenic acid, as was the case in this study. In turn, others obtained a similar relationship. According to Wojdyło et al. (2013, 2014) [7,33], in various quince fruit cultivars, the dominant phenolic acid was chlorogenic acid and its derivatives, i.e., neochlorogenic and cryptochlorogenic acid. Similarly, in fruits dried by various methods, chlorogenic acid dominated in the profile of these compounds [39]. In the study of quince fruit juice from 11 different cultivars, the highest concentration was found for chlorogenic acid and its derivatives, demonstrating a significant variation in its content that depended on the botanical variety [33].

The content of phenolic acids, in particular the content of chlorogenic acid, is crucial in the processing of quince fruit and largely determines the quality of products obtained from quince fruit, particularly juices [33]. These compounds are a natural substrate for enzymes (in particular, polyphenol oxidase) and significantly affect the processes of enzymatic oxidation and the color changes of processed products [8,9,33,44]. In addition, chlorogenic acid derivatives (mainly quinones) may oxidize other substances, e.g., flavan-3-ols, leading to the synthesis of colored compounds and increased browning of processed quince products [32,33]. According to Wojdyło et al. (2014) [33], the degree of browning in the case of quince fruit juices depends not only on the content of chlorogenic acid but also on the ratio of flavan-3-ols to hydroxycinnamic acids; therefore, juice production often requires the use of antioxidants, e.g., the addition of ascorbic acid [86,87].

**Figure 4:**
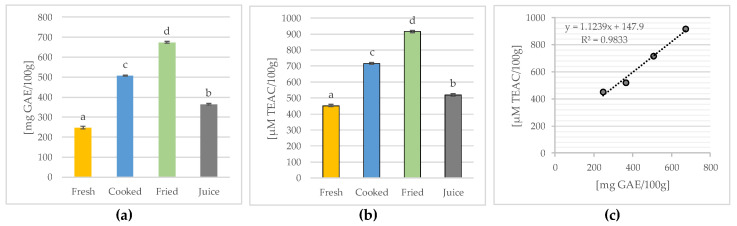
Total polyphenol content (**a**), antioxidant potential (**b**), and relation between total polyphenol content (mg GAE/100 g) and antioxidant potential (µM TEAC/100 g) (**c**) in fresh quince fruit and its products. Mean values (*n* = 3) marked in bars by different letters differ significantly (Duncan’s test, *p* ≤ 0.05). GAE—Gallic Acid Equivalent, TEAC—Trolox Equivalent Antioxidant Capacity.

Considering that the HPLC analysis determined the contents of the selected phenolic components and that the presented sum of carotenoids (Figure 3a), flavonoids (Figure 3b) and phenolic acids (Figure 3c) does not include all the of phenolic compounds, the total content of polyphenols was determined using the spectrophotometric method with the Folin–Ciocalteu reagent, an analytical procedure widely recognized and used in the literature for determining these components. The results are presented in Figure 4a.

Fresh quince fruits were characterized by a high content of total polyphenols (246.98 ± 6.76 mg GAE/100 g). This was confirmed by other authors, who demonstrated a similar content of these components (350 mg GAE/100 g) [7,11,26,39]. In other studies, the content of total polyphenols in the quince pulp was much lower (68.14 mg GAE/100 g) [54]; however, it was determined in the flesh without the skin, which has higher concentrations of these components than the flesh [26,85]. Varied results for both the flesh (71.03–158.89 mg GAE/100 g) and peel (140.12–202.92 mg GAE/100 g) of the fruit were also obtained by Stojanović et al. (2017) [84] (who mainly studied the effect of the quince variety on the content of these phytonutrients). Similarly, Szychowski et al. (2014) [12], by analyzing different quince varieties, showed differences in the content of total polyphenols, with higher values found in the skin (327–581 mg GAE/100 g) than in the fruit flesh (from 44.8–101 mg GAE/100 g). Generally, differences in the content of bioactive ingredients, including phenolics, are conditioned by the influence of various factors, i.e., genetic and environmental factors [11,12,55,56].

In the available literature, no studies on the effect of cooking, frying, and juicing on the total polyphenol content in quince fruits were found. Rather, the existing studies relate the comparison of this parameter in the final products [29,32,38] or in juices preserved with different methods [8,9,33]. The research showed a higher content of total phenolic compounds in heat-treated fruits, i.e., fried (674.21 ± 4.49 mg GAE/100 g) and boiled (507.79 ± 3.76 mg GAE/100 g) quince, and a lower content in the quince fruit juice (364.53 ± 3.76 mg GAE/100 g). In various quince fruit jams, Mir et al. (2016) [38] also observed an increase in the content of phenolic components in preserves compared to fresh quince fruit, which they explained by the effect of the heat treatment on the formation of new compounds, e.g., Maillard reaction products. Phenolic compounds (mainly glycosides) are concentrated in quince fruits, especially in the hydrophilic regions of cells (e.g., in vacuoles) or in the form of other water-soluble phytonutrients in the cytoplasm [38]. Therefore, it is likely that damage to the cell walls (during the heat treatment) insulating these components leads to an increase in their concentration in the products, while both the growth dynamics and the profile of these components depend primarily on the type of raw material and the technological treatment [38].

It should be emphasized that such a tendency was observed not only for the total polyphenols (Figure 4a), but also for the sum of the carotenoids (Figure 3a), flavonoids (Figure 3b), or phenolic acids (Figure 3c) identified by HPLC assay. It is also worth noting that for the sum of the determined carotenoids and phenolic acids, no significant differences were found between cooking and frying, but a significantly higher total flavonoid content was found in the fried fruits compared to the boiled ones, similar to the total polyphenols (Figure 4a) or antioxidant activity (Figure 4b).

In addition, a significant (*p* ≤ 0.05) correlation (R^2^ = 0.9833) was found between the total polyphenol content and antioxidant activity, both in fresh fruit and technologically treated fruit. This linear relationship is presented in Figure 4c. This relationship was also confirmed by other studies, both in the fresh fruit of different botanical quince varieties [7,12,21,86] and quince products such as jams [29,33], juices [33], or dried quince [39].

The differences in the content or profile of phenolic bioactive ingredients and in the antioxidant potential of quince fruits are explained primarily by the influence of the variety [7,12,33,56], but also the cultivation and growth conditions, the date of fruit harvest, and the degree of fruit maturity [7]. The type of tested plant tissue also has a very large impact on the content and mutual proportions of phytonutrients, with a much higher concentration of these components found in the skin than in the fruit [7,12,21,26,29,85]. Finally, the processing or preservation methods used [8,9], time, and storage conditions for quince products (e.g., juices) [33] also play important roles in shaping the profile of bioactive ingredients and their content and antioxidant activity in final quince fruit products.

In summary, the heat treatment of food (including fruit and vegetables) is usually associated with the loss of biologically active ingredients [88]. However, it can also lead to beneficial changes in the processed food, including the improvement of organoleptic properties and the sensory, textural, or nutritional value [76]. According to the conducted research, the heat treatment of quince fruit increased the content of bioactive ingredients, such as carotenoids or phenolic compounds, which was also confirmed by other authors’ studies [38]. According to the literature, the content of carotenoids [76,77,78,79,80], glucosinolates [89], or phenolic components [76,80] may increase in food products during heat treatments such as cooking, frying, baking [38,76,77,78,79,80], or microwave heating [90]. Many phenolic acids occur in fresh fruits, bounded in the fiber walls. After thermal processing, they are release to the free stage. This can explain the change in the total phenolic acids in quince after thermal processing [91,92]. On the other hand, in fresh fruits, flavonoids occur mostly in a glycoside form. After thermal processing, glycosides are lost to pure flavonoids [93]. This is explained by the ease of their release as a result of cellulose degradation in the cell walls, plant structure decomposition [94], and the hydrolysis of molecular complexes and the dissociation of bonds between food ingredients [95], which consequently leads to the release of polyphenols associated with dietary fiber [94] and increased levels of free phenolic compounds, among others [94,96]. In addition, under the influence of a heat treatment, new biologically active compounds are synthesized, including as a result of the Maillard reaction [97].

## 3. Materials and Methods

### 3.1. Materials

Quince fruit from a Polish farm (located in the Świętokrzyskie Voivodship), harvested in the autumn of 2021, was used for the study. The fruit was stored in refrigerated conditions (10 °C and a relative humidity of 85%) until the tests were carried out. For this study, 20 pieces of fruit of a similar weight (average 218.0 ± 17.6 g) were selected. The fruit was cleaned, washed, dried, peeled, deseeded, diced to a size of approximately 2 × 2 cm, divided into three parts, and subjected to cooking, frying in a pan without fat, and juicing to obtain juice.

The quince fruit was cooked in water (100 °C for 40 min) until the desired consistency (softness) was obtained, stirring occasionally. The cooked fruit was drained, slightly cooled, and rubbed on a laboratory sieve (S-3 Ø2.80 mm, BITLAB, Lublin, Poland) to obtain a relatively uniform consistency of mousse. It was then cooled down. The quince fruit was fried in a hot frying pan without fat for 40 min until the desired consistency was obtained, stirring frequently and adding small amounts of water (which was necessary to avoid burning). After frying and the evaporation of the water, the fruit was cooled. Fresh quince juice was obtained using a juicer (Zelmer Lumiere ZJE 1900X, Zelmer S.A., Warsaw, Poland). The control sample consisted of fresh fruit (Fresh) that was washed, cleaned, dried, and diced (without peeling or removing the cores). It should be noted that the results of the fresh quince fruit samples (Fresh) were published in the recent article by Najman et al. (2023) [39], and they are used for comparative purposes in this article.

### 3.2. Methods

#### 3.2.1. Dry Matter (d.m.), Moisture Content, Water Activity (a_w_), Soluble Solids Content (°Brix), pH, and Total Acidity (TA) of Quince and Its Products

The dry matter content was determined according to the AOAC method [98]. The weighing vessels were weighed, filled with quince fruit samples, reweighed, and dried (105 °C; 72 h) in a drying oven (SUP 200W, Wamed, Warsaw, Poland). The dry matter content (d.m.) was expressed as a percentage (%). The moisture content was calculated from the difference in mass and was also expressed as a percentage (%). Water activity (a_w_) was measured with an AquaLab Water Activity Meter (Decagon Devices. Inc., Pullman, WA, USA). The soluble solids content of the quince fruit and its products was measured using an Abbe Refractometer (ORT-1, Kern & Sohn GmbH, Balingen, Germany) at room temperature (20 °C), according to Polish Standard PN-EN 12143:2000 [99]. The soluble solids content (°Brix) was expressed as a percentage (%). The pH was measured by the potentiometric method at room temperature (20 °C), using a pH meter (Elmetron CP-511, Elmetron G.P., Zabrze, Poland). The total acidity was determined by the titration method, according to Polish Standard PN-EN 12147:2000 [100]. The total acidity (TA) was calculated into malic acid and expressed as g/100 g of the product.

All the measurements listed above were performed in three independent replicates.

#### 3.2.2. Color Parameters in L*a*b* Color Space of Quince and Its Products

The color parameters of fresh quince fruits and its products were achieved using a colorimeter (Konica Minolta CR-400, Konica Minolta, BSP, Warsaw, Poland) in the CIE LAB (L*a*b*) color space (*L*—lightness; *+a*—red; *−a*—green; *+b*—yellow; *−b*—blue), at room temperature (20 °C). The equipment was set up for illuminant D65 and an observer angle of 10°. It was calibrated using a standard white reflector plate, CR-A43 (*L* = 90.7; *a* = 0.9; *b* = −0.1). Three independent measurements were performed for each sample. 

According to Maskan (2001) [41], based on the obtained results, the browning index (BI) was also calculated, using equations:

(1) $$\mathrm{BI}=\frac{\left[100\left(x-0.31\right)\right]}{0.17}$$

where

(2) $$x=\frac{\left(a^{*}+1.75L^{*}\right)}{\left(5.654L^{*}+a^{*}-3.012b^{*}\right)}$$

#### 3.2.3. Tannin Content in Quince and Its Products

The determination of the tannin content in quince fruit and its products was carried out by the titration method in accordance with Ciszewska et al. (1975) [101], based on titration with a 0.05 M Na_2_S_2_O_3_ solution of the extracted tannins in the presence of 2% starch as an indicator. The details of the tannin extracts’ preparation and the analytical procedure were previously described in the work by Najman et al. (2023) [39]. The results were expressed as g/100 g of the product.

#### 3.2.4. Carotenoid Compounds of Quince and Its Products

The identification and determination of the content of selected carotenoids in quince fruit and its products were carried out in acetone extracts. In 10 mL tubes, 30.0 mg of the tested fruit samples were weighed, 10.0 mg of MgCO_3_ (magnesium carbonate) and 5.0 mL of acetone were added, vortexed (Wizard Advanced IR Vortex Mixer, VELP Scientifica Srl, Usmate Velate, Italy) for 60 s at 2000 rpm, incubated for 15 min (35 kHz; 20 °C) in an ultrasonic bath (Bandelin Sonorex RK 255, BANDELIN Electronic, Berlin, Germany), and centrifuged (MPW-380 R, MPW Med. Instruments, Poland, Warsaw) for 15 min (4 °C; 10,000 rpm). The obtained supernatant was transferred into HPLC vials and analyzed by the HPLC method (Shim-pol HPLC-set (Warsaw, Poland), using two LC-20AD pumps, a CMB-20A system controller, an SIL-20AC autosampler, an SPD-20AV/VIS detector, and a CTD-20A oven) in accordance with Ponder et al. (2021) [81].

The separation of the carotenoid compounds was carried out using a Max-RP 80A chromatographic column (4.60 × 250 mm). The following analysis conditions were used: a mobile acetone:*n*-hexane (5:95) phase, a flow rate of 1.5 mL/min, an analysis time of 30 min; detection at λ = 445–480 nm. External standards, i.e., lutein, zeaxanthin, chlorophyll a, chlorophyll b, and β-carotene with a purity of 99.9% were used, and the content of carotenoids was expressed as mg/100 g of the product.

#### 3.2.5. Phenolic Compounds (Flavonoids and Phenolic Acids) of Quince and Its Products

The identification and determination of the content of selected flavonoids and phenolic acids in quince fruit and its products were carried out in methanol extracts. In 10.0 mL tubes, 100.0 mg of the fruit samples and 5.0 mL of 80% methanol were added, vortexed (Wizard Advanced IR Vortex Mixer, VELP Scientifica Srl, Usmate Velate, Italy) for 60 s at 2000 rpm, incubated for 15 min (35 kHz; 20 °C) in an ultrasonic bath (Bandelin Sonorex RK 255, BANDELIN Electronic, Berlin, Germany), and centrifuged (MPW-380 R, MPW Med. Instruments, Poland, Warsaw) for 15 min (4 °C; 10,000 rpm). The obtained supernatant was transferred into HPLC vials and analyzed by the HPLC method (Shimadzu HPLC-set (USA Manufacturing Inc., USA Manufacturing Inc., Gaithersburg, MD, USA), using two LC-20AD pumps, CMB-20A and CTD-20AC system controllers, an SIL-20AC autosampler, an SPD-20AV UV/VIS detector, and a CTD-20A oven in accordance with Hallmann et al. (2017) [102].

The separation of phenolic compounds was carried out using a Synergi Fusion-RP 80i chromatographic column (250 × 4.60 mm). The following analysis conditions were used: a two-phase flow gradient of acetonitrile/deionized water (55% and 10%); pH 3.00, a flow rate of 1.0 mL/min, an analysis time of 38 min; detection at λ = 250–370 nm. External standards, i.e., catechin, epigallocatechin, rutoside-3-*O*-quercetin, and quercetin and gallic, chlorogenic, *p*-coumaric, ferulic, and caffeic acids with a purity of 99.5–99.9% were used, and the contents of the selected flavonoids and phenolic acids were expressed as mg/100 g of the product.

#### 3.2.6. Total Polyphenol Content and Antioxidant Potential of Quince and Its Products

The determination of the total polyphenol content and the antioxidant potential of the quince fruit and its products were carried out in water extracts. In 50.0 mL Falcone tubes, 500.0 mg of the fruit samples and 40.0 mL of distilled water were added, vortexed (Wizard Advanced IR Vortex Mixer, VELP Scientifica Srl, Usmate Velate, Italy) for 60 s at 2000 rpm, incubated for 60 min (60 °C; 200 rpm) in an a shaking incubator (IKA KS 4000i Control, IKA^®^ Poland Ltd., Warsaw, Poland), and centrifuged (MPW-380 R, MPW Med. Instruments, Poland, Warsaw) for 15 min (4 °C; 10,000 rpm). The obtained supernatant was used to determine the total polyphenol content and antioxidant activity by the colorimetric, spectrophotometric method (UV/Vis UV-6100A, Metash Instruments Co., Ltd., Shanghai, China).

The total polyphenol content was determined using the Folin–Ciocalteu reagent in accordance with the Singleton and Rossi (1965) [103] method. After 60 min of incubation (20 °C; without access to light), the absorbance of the mixture (1.0 mL of aqueous extract of quince fruit sample; 2.5 mL of Folin–Ciocalteu reagent; 5.0 mL of 20% Na_2_CO_3_ (sodium carbonate) in 41.5 mL of distilled water) was measured at λ = 750 nm. Based on the absorbance results and the standard curve (y = 2.1297x + 0.1314, R^2^ = 0.9994) for gallic acid, the results were calculated and expressed as mg GAE/100 g of product (GAE—gallic acid equivalent).

The antioxidant potential was determined using the cation radical ABTS^+•^ (2,2’-azino-bis 3-ethylbenzothiazolin-6-sulfonic acid) in accordance with the method of Re et al. (1999) [104]. After 6 min of incubation (20 °C), the absorbance of the mixture (1.5 mL of aqueous extract of quince fruit sample and 3.0 mL radical cations ABTS^+•^ in PBS solution (PBS—phosphate buffer solution) was measured at λ = 734 nm. Based on the absorbance results and the standard curve (y = −5.6017x + 0.7134; R^2^ = 0.9998) for Trolox, the results were calculated and expressed as µM TEAC/100 g of the product (TEAC—Trolox equivalent antioxidant capacity).

#### 3.2.7. Chemicals

All chemicals used for the tests, including the total acidity (TA), carotenoid and polyphenolic compounds (HPLC method), total polyphenol content, antioxidant potential, and the tannin content were of analytical or HPLC grade (purity of 99.5–99.9%) and were from Sigma-Aldrich (Poznań, Poland).

#### 3.2.8. Statistical Analysis

The obtained results are presented in tables or figures as mean values ± standard deviation (SD) of three to six independent repetitions. The statistical analyses were performed using Statistica 13.0 (Tibco Software Inc., Palo Alto, CA, USA). To assess the similarities and differences between the samples, a one-way ANOVA (Duncan’s post hoc test) was performed. Differences were considered statistically significant at *p* ≤ 0.05.

## 4. Conclusions

The conducted research is important from both the cognitive and application perspectives of the obtained results. They demonstrate the possibilities of using quince fruits, which are unsuitable for direct consumption due to their sour, tart taste and hard texture, and provide knowledge on the comparison of the content of bioactive compounds and the physicochemical properties of preserves obtained from these fruits. The highest bioactive properties, i.e., the content of carotenoids, flavonoids, and polyphenolic compounds, and the highest antioxidant potential were found in the heat-treated quince fruit, in particular the fried fruit (166.12 ± 0.62 mg/100 g; 67.49 ± 0.66 mg/100 g; 674.21 ± 4.49 mg GAE/100 g; 916.16 ± 6.65 µM TEAC/100 g, respectively). These properties and the antioxidant potential were found to be lower in freshly squeezed quince fruit juice (141.88 ± 0.62 mg/100 g; 40.07 ± 1.91 mg/100 g; 364.53 ± 3.76 mg GAE/100 g; 520.76 ± 8.56 µM TEAC/100 g; respectively). The obtained preparations were characterized by a high content of the tested bioactive compounds, higher than their level in raw fruit (44.98 ± 0.18 mg/100 g; 16.92 ± 0.13 mg/100 g; 246.98 ± 6.76 mg GAE/100 g; 452.41 ± 6.50 µM TEAC/100 g, respectively, for carotenoids, flavonoids, total polyphenol content, and antioxidant activity), indicating the desirability of their use not only in the home production of preserves but also in industrial production, introducing products that are a source of many valuable bioactive compounds to the market and diversifying the offer of fruit concentrates produced thus far. The choice of the appropriate technological processing of quince fruit can be the starting point for the development of many new functional products with their addition in various forms, providing the producers of functional food with a wide range of opportunities to expand the range of products manufactured thus far.

## Figures and Tables

**Table 1:** Color parameters in L*a*b* space of fresh quince fruits and quince products.

Color Parameter	Fresh	Cooked	Fried	Juice
L* (lightness)	82.11 ± 3.48 ^c^	71.93 ± 0.11 ^b^	73.74 ± 1.27 ^b^	56.47 ± 0.76 ^a^
a* (redness)	11.54 ± 0.59 ^b^	7.43 ± 0.01 ^a^	10.68 ± 0.62 ^b^	13.99 ± 0.02 ^c^
b* (yellowness)	58.76 ± 2.01 ^d^	45.23 ± 0.15 ^b^	51.62 ± 1.11 ^c^	41.61 ± 0.04 ^a^
BI (browning index)	124.84 ± 15.08 ^b^	89.78 ± 0.31 ^a^	120.60 ± 7.73 ^b^	155.36 ± 2.92 ^c^

Mean values ± SD (*n* = 3) marked by different letters in the same line differ significantly (Duncan’s test, *p* ≤ 0.05).

**Table 2:** Physicochemical characteristics of fresh quince fruit and its products.

Parameter	Fresh	Cooked	Fried	Juice
Dry matter (%)	18.60 ± 0.16 ^c^	15.29 ± 0.38 ^a^	21.90 ± 0.00 ^d^	16.92 ± 0.07 ^b^
Moisture (%)	81.40 ± 0.16 ^b^	84.71 ± 0.38 ^c^	78.10 ± 0.00 ^a^	83.08 ± 0.07 ^b^
Water activity (a_w_)	0.98 ± 0.00 ^a^	0.99 ± 0.00 ^a^	0.98 ± 0.00 ^a^	0.97 ± 0.00 ^a^
°Brix (%)	12.50 ± 0.50 ^a^	12.00 ± 0.50 ^a^	16.83 ± 0.58 ^c^	15.17 ± 0.29 ^b^
pH	4.19 ± 0.03 ^c^	3.78 ± 0.01 ^a^	3.73 ± 0.01 ^a^	3.93 ± 0.01 ^b^
Total acidity (g/100 g)	0.26 ± 0.00 ^a^	0.37 ± 0.01 ^b^	0.55 ± 0.03 ^c^	0.40 ± 0.02 ^b^

Mean values ± SD (*n* = 3) marked by different letters in the same line differ significantly (Duncan’s test, *p* ≤ 0.05).

**Table 3:** Selected carotenoid compounds in fresh quince fruit and its products.

Carotenoids	Fresh	Cooked	Fried	Juice
Lutein (mg/100 g)	3.55 ± 0.06 ^a^	13.16 ± 0.08 ^c^	13.84 ± 0.06 ^c^	8.41 ± 0.09 ^b^
Zeaxanthin (mg/100 g)	1.39 ± 0.00 ^a^	5.56 ± 0.00 ^b^	5.56 ± 0.00 ^b^	5.56 ± 0.00 ^b^
Chlorophyll a (mg/100 g)	14.22 ± 0.13 ^a^	47.90 ± 0.47 ^c^	48.99 ± 0.61 ^c^	37.72 ± 0.21 ^b^
Chlorophyll b (mg/100 g)	12.22 ± 0.16 ^a^	43.93 ± 0.83 ^c^	43.34 ± 0.78 ^c^	35.87 ± 0.43 ^b^
β-carotene (mg/100 g)	13.61 ± 0.00 ^a^	54.38 ± 0.01 ^b^	54.38 ± 0.01 ^b^	54.33 ± 0.00 ^b^

Mean values ± SD (*n* = 3) marked by different letters in the same line differ significantly (Duncan’s test, *p* ≤ 0.05).

**Table 4:** Selected phenolic compounds in fresh quince fruit and its products.

Phenolic Compounds	Fresh	Cooked	Fried	Juice
Selected flavonoids				
Catechin (mg/100 g)	3.33 ± 0.09 ^a^	11.82 ± 0.25 ^d^	10.59 ± 0.03 ^c^	7.94 ± 0.21 ^b^
Epigallocatechin (mg/100 g)	8.04 ± 0.05 ^a^	30.15 ± 0.74 ^c^	37.00 ± 0.78 ^d^	13.63 ± 0.81 ^b^
Rutoside-3-*O*-quercetin (mg/100 g)	4.60 ± 0.26 ^a^	17.88 ± 0.81 ^c^	16.41 ± 0.76 ^b^	16.20 ± 0.83 ^b^
Quercetin (mg/100 g)	0.95 ± 0.01 ^a^	3.48 ± 0.10 ^c^	3.50 ± 0.12 ^c^	2.30 ± 0.07 ^b^
Selected phenolic acids				
Gallic acid (mg/100 g)	1.10 ± 0.02 ^a^	15.71 ± 0.12 ^d^	14.68 ± 0.07 ^c^	5.63 ± 0.11 ^b^
Chlorogenic acid (mg/100 g)	14.47 ± 0.14 ^a^	205.74 ± 1.12 ^c^	209.58 ± 2.32 ^c^	48.27 ± 1.57 ^b^
Caffeic acid (mg/100 g)	0.61 ± 0.00 ^a^	9.86 ± 0.10 ^d^	2.91 ± 0.06 ^b^	4.02 ± 0.04 ^c^
*p*-coumaric (mg/100 g)	0.39 ± 0.00 ^a^	6.67 ± 0.22 ^b^	6.31 ± 0.41 ^b^	6.17 ± 0.17 ^b^
Ferulic acid (mg/100 g)	0.31 ± 0.00 ^a^	4.99 ± 0.08 ^c^	2.01 ± 0.07 ^b^	2.08 ± 0.08 ^b^

Mean values ± SD (*n* = 3) marked by different letters in the same line differ significantly (Duncan’s test, *p* ≤ 0.05).

## Data Availability

Not applicable.
